# Trace metals and micronutrients in bone tissues of the red fox *Vulpes vulpes* (L., 1758)

**DOI:** 10.1007/s13364-012-0073-1

**Published:** 2012-02-10

**Authors:** Natalia Lanocha, Elzbieta Kalisinska, Danuta I. Kosik-Bogacka, Halina Budis, Kinga Noga-Deren

**Affiliations:** 1 Department of Biology and Medical Parasitology, Pomeranian Medical University of Szczecin, Powstancow Wielkopolskich 72, 70-111 Szczecin, Poland; 2 Department of Preclinical Conservative Dentistry and Preclinical Endodontics, Pomeranian Medical University of Szczecin, Powstancow Wielkopolskich 72, 70-111 Szczecin, Poland

**Keywords:** Red fox, Bioindicator, Bone tissue, Trace elements

## Abstract

In this study we determined the levels of trace elements (zinc, copper, lead, cadmium and mercury) in three layers of bones of the hip joint (cartilage, compact bone and spongy bone) of 30 red foxes (*Vulpes vulpes*) from north-western Poland. Concentrations of Cu, Zn, Pb and Cd were determined by atomic absorption spectrophotometry (ICP-AES) in inductively coupled argon plasma using a Perkin-Elmer Optima 2000 DV. Determination of Hg concentration was performed by atomic absorption spectroscopy. In cartilage, compact bone and spongy bone samples from the red fox, median concentrations of the metals studied could be arranged in the following descending series: Zn > Cu > Pb > Cd > Hg, the values ranging from 142 to 0.002 mg/kg dw. There was a significant difference in Cu concentrations, among all the materials analyzed, with much more Cu found in spongy bone than in compact bone. Significant differences were also noted in the case of Hg concentrations in cartilage with compact bone and the spongy bone, and between concentrations of this metal in compact bone and spongy bone. In males, the concentration of Hg in spongy bone was greater than in females. Younger foxes had a higher concentration of this metal in cartilage than adults. The strongest synergistic relationships were observed in spongy bone between the Zn and Cu, Zn and Cd, as well as between Cu and Cd. Statistically significant antagonistic relationships were detected between zinc and lead in compact bone. In addition to monitoring studies conducted on the abiotic environment, an urgent need exists for long-term monitoring of concentrations of heavy metals with long-term effects on living organisms. An important addition is provided by biomonitoring studies on domesticated and free-living mammals, including Canidae.

## Introduction

Representatives of Canidae, namely the red fox *Vulpes vulpes* (Linnaeus, 1758), the domesticated dog *Canis lupus familiaris* (Linnaeus, 1758), the wild raccoon dog *Nyctereutes procyonoides* (Gray 1834), and the wolf *Canis lupus* (Linnaeus, 1758), are all common objects of ecotoxicological studies (Kalisinska et al. 2009; 2011; Millan et al. 2008; Shore et al. 2001).

The fox belongs to a group of hunted mammals commonly found in Eurasia and North America. It is a predator preferring food of animal origin including rodents, birds and small invertebrates (beetles, grubs, earthworms) (Kidawa and Kowalczyk 2011). The species occupies a high position in the food pyramid and accumulates ingested substances. It also exhibits a measurable response to environmental contaminants, including heavy metals (Kalisinska et al. 2009; 2011; Lopez-Alonso et al. 2007).

Many reports mention the neuro-, nephro- or hepatotoxic effects of exposure to trace elements, but publications on the accumulation of trace elements (including zinc, copper, lead, cadmium and mercury) in cartilage and bone elements of human and animal joints are much less numerous (Brodziak-Dopierala et al. 2007; Jankovska et al. 2010; Kalisinska et al. 2007; Kwapulinski et al. 1995; Piskorova et al. 2003).

We examined bone tissues of the red fox in a risk assessment of exposure to trace elements, as they are subject to slow transfer of some metals in the body. Due to its characteristics and long renewal time, this tissue may reflect levels of chronic exposure and could be the basis of indirect environmental assessment (Brodziak-Dopierala et al. 2007; 2009; Zaichick and Zaichick 2009; 2010; Zaichick et al. 2011).

### Zinc (Zn) and copper (Cu)

Zinc and copper are involved in the formation and metabolism of bone tissue (Honda et al. 1997; Nielsen and Milne 2004; Senczuk 2006; Yamaguchi 1998).

Zinc is essential for the correct ossification and mineralization of bones, especially in the basal part of the femur. It is a cofactor for the enzyme affecting the synthesis of various ossein components and plays a role in the regulation of bone resorption (Machalinski et al. 1996; NRC 1980; Puzanowska-Tarasiewicz et al. 2009; Smrcka 2005). Both too high and too low concentrations of Zn contribute to the gradual reduction of bone mass and reduce the concentration of calcium ions in bones and blood serum (Charles et al. 2001).

Copper in mammals is involved in the process of the hardening of collagen, hair keratinization and also normalizes the deposition of calcium and phosphorus in bones. Cooper inhibits bone resorption, which may occur due to a reduction in prostaglandin synthesis (Senczuk 2006). Copper deficiency leads to reduced bone mass, resulting in a decrease in its mechanical strength and subsequent fractures.

Studies on Cu and Zn supplementation in humans and animals indicate that a deficiency of these micronutrients leads to osteoporosis-like changes (Nielsen and Milne 2004).

### Lead (Pb), cadmium (Cd) and mercury (Hg)

The presence of Pb, Cd and Hg have been detected in all tissues of mammals, and even minimum concentrations have been observed to cause metabolic disturbances, thus reducing physical efficiency, weakening immune and enzymatic processes, and leading to many diseases and sometimes death.

Lead toxicity is manifested in anemia, impaired nervous system and kidney function and changes in bones such as decreased bone mass (osteopenia) and delayed healing of fractures (Gerhardsson et al. 2005; Kjellstrom 1992; Wiechula et al. 2008). Accumulation of Pb in bone, in contrast to solid organs, increases with age (Jurkiewicz et al. 2004).

Cadmium has mainly nephro- and hepatotoxic properties. The osteotoxic action of Cd was described in Japan as early as 1960 in 90% of older women (after menopause and many pregnancies) living in areas contaminated with Pb and Zn ores. The disease was called "Itai-Itai" (ouch-ouch disease). First, it was observed in the Japanese population of the Jinzu River overflow area, consuming mainly rice grown in fields fertilized with silt derived from local plants (Starek 2007; Zhu et al. 2004). The concentration of Cd in that soil was 8 mg/kg dw, and in rice up to 2.7 mg/kg. The patients had osteomalacia resistant to vitamin D, accompanied by severe pain around the sacrum, the lower limbs and ribs, spontaneous fractures, as well as proteinuria, glycosuria and decreased sodium reabsorption (Bernard 2008; Horiguchi et al. 2010; Jarup and Akesson 2009; Umemura and Wako 2006).

Mercury is a potent neurotoxin, primarily disrupting the function of the central and peripheral nervous system (Scheuhammer et al. 2008). However, individual studies have drawn attention to the deposition of this metal in skeletal elements (Yoo et al. 2002; Zaichick and Zaichick 2010; Zaichick et al. 2011).

The determination of toxic element concentrations in living organisms is one of the basic methods of indirect assessment of environmental pollution. Ecotoxicological studies in Poland and the rest of the world are usually carried out on the liver and kidneys of warm-blooded vertebrates. However, there has been an increasing number of biomonitoring studies on bones. Apart from environmental studies, a large need exists for the monitoring of concentrations of heavy metals in humans, which can be supplemented with biomonitoring of both domesticated animals (such as the pig, sheep, cattle, horse and dog) and wild animals (e.g., fox, boar, roe deer and deer) (Kalisinska et al. 2009; Lanocha et al. 2009; Lazarus et al. 2008; Liu 2003).

The aim of this study was to determine the concentrations of five elements: two micronutrients (Zn and Cu) and three toxic trace metals (Pb, Cd and Hg) in three types of biological materials derived from the bones of the red fox *V. vulpes*, and to determine intraspecific differences between the concentrations of trace elements in cartilage, spongy bone and compact bone.

## Materials and methods

### Study area

The material was collected in north-western Poland, in the West Pomeranian province including its capital, Szczecin. Most of the province's area is agricultural (38%) and forested (35%), and several percent of the area is covered by water (numerous lakes, rivers, the Odra estuary with Dabie Lake, and Szczecin Lagoon) (http://www.stat.gov.pl/cps/rde/xbcr/szczec/ASSETS_przegl_2.pdf).

### Material

The material was collected in 2008–2009. Altogether it consisted of 30 foxes from six districts of the West Pomeranian province (municipalities of Szczecin, Choszczno, Stargard, Gryfice, Kamien Pomorski and Mysliborz — three, eight, seven, one, two, and two specimens, respectively, and seven remaining foxes from the Provincial Veterinary Inspectorate in Szczecin). In Poland, the fox is included in the list of animals for hunting (*Journal of Law* 2005, no 45, pos. 433), and according to the Minister of Environment, it may be hunted from 1st July to 31st March (*Journal of Law* 2005, no 48, pos. 459).

The acquisition of biological material from the foxes was approved by the Local Ethics Committee for Research on Animals in Szczecin (Poland).

### Fox age determination

Fox age categories were based on the examination of one single-root lower canine, with preserved anatomical crown, from 30 foxes. The teeth were placed immediately after extraction in distilled water, and then dried. In order to obtain pantomographic images, all the teeth were glued on cardboard sheets. Radiographs were performed on a Cranex Ceph's Soredex digital pantomogram from a distance of 120 cm (60 kV, 10 mA s). Measurements of linear parameters were performed using digital radiography DIGORA 2.1 software (Soredex-Orion, Helsinki, Finland).

They included the total width of the tooth (TW) and width of the pulp chamber (WC). According to the work of Knowlton and Whittemore (2001), the width of the canine pulp chamber was measured at a standard distance of 15 mm from the root apex. Canine width index (CWI) was calculated as the ratio of the width of the pulp chamber to the overall width of the lower canine, which allowed the division of subjects into two age categories (adultus [ad]; immaturus [im]). It was assumed that immature foxes were in the range of CWI from 0.20 to 0.50, and adults from 0.05 to 0.20 (Cavallini and Santini 1995). Among the specimens collected for analysis, some teeth had a very large pulp chamber and others very narrow. Following CWI values it was determined that the examined group included 18 im and 12 ad foxes, respectively (Table 1).

**Table 1:** Sizes of mandibular canine teeth from immature (im) and adult (ad) foxes

Age category and number of specimens (*n*)	Parameter	WC	TW	CWI
im (*n* = 18)	AM ± SD	1.49 ± 0.58	4.95 ± 0.91	0.29 ± 0.10
	Med	1.39	5.24	0.28
	Range	0.72–3.0	2.5–5.7	0.19–0.60
	CV	39.3	18.5	35.4
ad (*n* = 12)	AM ± SD	0.65 ± 0.33	5.64 ± 0.70	0.11 ± 0.05
	Med	0.50	5.63	0.09
	Range	51.1	12.4	47.9
	CV	0.20–1.27	4.50–7.27	0.03–0.19
Total (*n* = 30)	AM ± SD	1.15 ± 0.65	5.23 ± 0.89	0.22 ± 0.13
	Med	1.13	5.25	0.23
	Range	0.2–3.0	2.50–7.27	0.03–0.60
	CV	56.0	17.0	57.4

*WC* width of the pulp chamber, *TW* total width, *CWI* canine width index, *AM* arithmetic mean, *Med* median, *SD* standard deviation, *CV* coefficient of variation in %

### Preparation of material for analysis of bone tissues

The head, neck and part of the femoral shaft were collected from the foxes using a glass tool. Chemical analysis was performed on three materials: cartilage, compact bone and spongy bone with directly adjacent compact bone. Bone tissue was dried to constant weight at 55°C and 105°C in drying oven with natural convection, ED 53 (Binder GmbH, Germany). This procedure was used to determine the water content (gravimetric method). Dried samples were ground in an agate mortar (Sigma-Aldrich, Poland).

### Determination of zinc, copper, cadmium and lead

The samples were divided into doses, weighing from 0.5 to 1.0 g. Bone tissue was mineralized by wet digestion using a Velp Scientifica mineralizer (Italy) (Kalisinska et al. 2007).

Concentrations of Zn, Cu, Pb and Cd were determined by atomic absorption spectrophotometry (ICP-AES) in inductively coupled argon plasma, using a Perkin-Elmer Optima 2000 DV. The device’s limits of detection for Zn, Cu, Pb, Cd were 0.2, 0.4, 1 and 0.1 μg/L, respectively.

### Determination of mercury

Total mercury (THg) concentrations were determined in samples dried at 55°C, using atomic absorption spectroscopy. The assays were run in an AMA 254 mercury analyzer (Altach Ltd, Czech Republic). For the analysis, we collected from 100 to 300 mg of the sample and then placed it in a nickel nacelle in which it was automatically weighed and dried. The sample was thermally decomposed in a stream of oxygen to obtain the gaseous form, and its degradation products were transferred to an amalgamator for the selective off take of Hg. After determination of the parameters of measurement, Hg vapor was released from the amalgamator by brief heating. The amount of released Hg was measured by atomic absorption (silicon UV diode detector in the AMA 254 analyzer) at a wavelength of 254 nm, in the arrangement of two measuring cells. The limit of detection for this method is 0.01 ng/100 mg of Hg in the sample. For each sample, two or three repetitions were performed, and the statistical analysis used the average of the data, expressed in mg/kg dry mass (dw).

### Validation of analytical proceedings

The reliability of the analytical procedure was controlled by the determination of elements in two reference materials with known concentrations: NIST SRM 1486 Bone Meal, and IAEA-407 Trace Elements and Methylmercury in Fish (National Institute of Standards and Technology [NIST] and the International Atomic Energy Agency [IAEA]). Concentrations of metals in the reference materials provided by the manufacturers and our own determinations are shown in Table 2.

**Table 2:** Concentrations of selected elements in the certified reference materials in mg/kg dry weight

Metal	Bone Meal SRM NIST 1486		OD/RV (%)	Fish Tissue IAEA-407		OD/RV (%)
	RV	OD (*n* = 7)		RV	OD (*n* = 8)	
Zn	147.0 ± 16.0	132.4 ± 4.1	90.0	67.1	65.8 ± 3.8	98.1
Cu	0.80^a^	0.74 ± 0.01	92.5	3.28	3.12 ± 0.28	95.1
Pb	1.335 ± 0.014	1.190 ± 0.306	89.1	0.12	0.11 ± 0.03	91.7
Cd	0.003^a^	0.0020 ± 0.0002	66.7	0.189	0.176 ± 0.010	93.1
Hg	–	–	–	0.222	0.237 ± 0.002	106.8

*RV* reference value, *OD* own determination

^a^Estimated value

### Statistical analysis

The analysis used Statistica 9.0. StatSoft software. In order to determine compliance with the expected normal distribution of results, we used a Kolmogorov–Smirnov test with Lillefors correction (*p* < 0.05). In order to compare the impact of various environmental factors on the concentration of metals in the bone material marrow test, we used a Kruskall–Wallis test, and in the case of significant differences, a Mann–Whitney *U*-test (*p* < 0.05).

In addition, we determined the Spearman rank correlation coefficients between trace elements in different parts of the hip joint (cartilage, compact bone, spongy bone, cartilage with compact bone).

## Results

Basic data on the concentrations of metals in the fox bone material is presented in Table 3. Because two samples (compact bone and spongy bone) derived from two individuals exceeded Cu concentration found in the other samples many times, we also conducted statistical analysis of Cu without taking these samples into account. The concentration of Cu in these samples was 20.35 mg/kg dw in spongy bone and 37.5 mg/kg dw in compact bone. In other samples, the concentration of this metal did not exceed 2.5 mg/kg dw. Both specimens came from urban areas and had fed mostly from trash cans, which may be the reason for such high Cu levels.

**Table 3:** Concentrations of trace elements (in mg/kg dry weight) in four types of bone material coming from fox in the vicinity of Szczecin

Metal	Parameter	Cartilage	Compact bone		Cartilage with adjacent compact bone		Spongy bone		Significance K–W
Zn	AM ± SD	134.3 ± 55.6	125.1 ± 56.3		129.7 ± 55.0		111.04 ± 48.74		NS
	Med	141.8	105.9		130.4		116.31		
	range	19.6–219.8	46.2–296.2		19.6–296.2		11.21–219.51		
	CV	41.4	45.0	42.9			43.9		
Cu^a^			*n* = 30	*n* = 29	*n* = 30	*n* = 29	*n* = 30	*n* = 29	*p* < 0.05
	AM ± SD	1.80 ± 2.70	2.01 ± 6.72	0.77 ± 0.46	1.89 ± 5.07	1.29 ± 2.0	1.17 ± 3.64	0.50 ± 0.35	
	Med	0.88	0.72	0.69	0.82	0.80	0.43	0.41	
	range	0.10–9.66	0.13–37.5	0.13–2.18	0.10–34.49	0.10–9.66	0.07–20.36	0.07–1.18	
	CV	149.6	336.6	59.7	267.4	154.4	312.3	69.6	
Pb	AM ± SD	1.738 ± 2.325	0.978 ± 1.153		1.36 ± 1.86		1.49 ± 1.83		NS
	Med	0.788	0.447		0.469		0.610		
	range	0.165–11.017	0.150–4.354		0.150–11.017		0.069–6.147		
	CV	133.8	117.9		136.9		123.0		
Cd	AM ± SD	0.137 ± 0.055	0.108 ± 0.073		0.123 ± 0.060		0.142 ± 0.07		NS
	Med	0.163	0.125		0.150		0.169		
	range	0.028–0.198	0.002–0.226		0.002–0.226		0.034–0.260		
	CV	40.0	67.7		53.6		49.2		
Hg	AM ± SD	0.0060 ± 0.0052	0.0054 ± 0.0047		0.0057 ± 0.0049		0.0029 ± 0.0023		*p* < 0.05
	Med	0.0044	0.0037		0.0038		0.0019		
	range	0.0016–0.0223	0.0012–0.0226		0.0011–0.0226		0.0013–0.0105		
	CV	87.2	87.2		86.7		77.2		

*AM* arithmetic mean, *SD* standard deviation, *Med* median, *CV* coefficient of variation (in %); *K–W* Kruskall–Wallis test, *p* level of significance, *NS* difference non-significant

^a^Analysis for all specimens (*n* = 30), after removing samples with exceptionally high Cu concentration

The distribution of empirical data on the concentrations of Pb, Cd and Hg in the cartilage, compact bone and spongy bone, and Zn in the fox compact bone, diverged from the expected normal distribution, and was examined using a Kolmogorov–Smirnov test (*p* > 0.05) with Lillefors correction (*p* < 0.05). For the concentration of Cu, the distribution of all results was not consistent with the expected normal distribution, but after removal of the two aforementioned exceptional samples, the distribution of the remaining results was consistent with the expected normal distribution (Table 3).

Among the micronutrients, Zn had the highest concentration in samples obtained from the examined fox bones, with the median values ranging from about 100 to 140 mg/kg dw, depending on the type of material. However, the differences between Zn concentrations were not significant (Table 3).

The average concentration of Cu in different types of samples ranged from about 0.40 to 0.90 mg/kg dw, and the Kolmogorov–Smirnov test revealed the existence of a statistically significant difference (*p* < 0.05). The highest concentration of Cu was observed in cartilage (0.88 mg/kg), and it was clearly higher compared to compact bone, cartilage, compact bone and spongy bone by 28%, 10% and 115%, respectively.

In the group of highly toxic metals, Pb had the greatest levels, with the median value ranging from about 0.45 mg/kg (compact bone) to about 0.80 mg/kg dw (in cartilage), but with no statistically confirmed differences between the types of samples analyzed (Table 3). The maximum Pb concentration in cartilage exceeded 10 mg/kg, and in half of the samples was greater than 1 mg/kg dw. In other types of samples maximum Pb concentrations were not greater than 4.40 and 6.15 mg/kg in the compact bone and spongy bone, respectively (Table 3).

Mean Cd concentration in the analyzed materials ranged from 0.125 mg/kg (compact bone) to about 0.170 mg/kg (spongy bone), and the differences between them were statistically significant. The highest Cd concentration was found in the spongy bone (0.169 mg/kg dw) and was higher compared to levels in cartilage, compact bone and cartilage with adjacent compact bone by 4%, 35% and 13%, respectively.

The average Hg concentration in the various types of samples ranged from about 0.002 (in the spongy bone) to more than 0.004 mg/kg dw (in cartilage) and the Mann–Whitney *U*-test revealed statistically significant differences (*p* < 0.05). Significant differences were found between the concentration of this metal in the cartilage with the adjacent compact bone and in spongy bone. Such differences also existed between the Hg concentration in the compact bone and spongy bone, with about 50% higher concentration of this metal in the compact bone underlying the cartilage (Tables 3 and 4). Maximum Hg concentration in the compact bone reached 0.0226 mg/kg, and in 17% of the samples it was greater than 0.0100 mg/kg dw.

**Table 4:** Significance of differences between metal concentrations in different fox bone materials

Metal	Parameter	C vs. CB	C vs. SB	CB vs. SB	C + CB vs. SB
Zn	*U*	NS	NS	NS	NS
*n* = 30	*p*				
Cu	*U*	297.0	217.0	293.0	510.0
*n* = 29	*p*	0.04	0.001	0.03	0.001
Pb	*U*	NS	NS	NS	NS
*n* = 30	*p*				
Cd	*U*	NS	NS	300.0	NS
*n* = 30	*p*			0,03	
Hg	*U*	NS	210.0	243.0	451.0
*n* = 30	*p*		0.001	0.002	0.002

*C* cartilage, *CB* compact bone, *C + CB* cartilage with compact bone, *SB* spongy bone, *U* Mann–Whitney *U*-test, *p* level of significance

In fox cartilage, compact bone and spongy bone, concentrations of the examined metals can be arranged in the following order: Zn > Cu > Pb > Cd > Hg.

Comparative analysis, which included sex, showed that between males and females, in principle, there were no statistically proven differences in the concentrations of metals determined in the corresponding bone material. Mercury in the spongy bone is an exception, as its concentration in females was 0.0017 mg/kg dw and was over 70% lower compared to males, where it was on average 0.0029 mg/kg dw (*U*-test = 63.0, *p* < 0.05).

In addition, a comparison of metal concentrations in the corresponding bone material was carried out between foxes representing two age categories — immature foxes (im) and the adults (ad) (Table 5). Only in the case of Hg in cartilage was there a confirmed statistical difference (*U*-test = 63.0, *p* < 0.05); the average Hg concentration in young foxes was about 100% higher than in adults (0.0054 and 0.0027 mg/kg dw, respectively). In addition, the cartilage Pb concentration in the ad group was about 118% higher than in the im group (0.516 and 1.124 mg/kg dw), but this difference was not statistically confirmed (*p* = 0.98).

**Table 5:** Comparison of metal concentrations in analagous materials between immature and adult foxes

Immature, *n* = 18						
Cartilage	AM ± SD	136.6 ± 52.8	1.90 ± 1.34	1.876 ± 2.755	0.147 ± 0.129	0.0069 ± 0.0053
	Med	136.6	2.43	0.516	0.166	0.0054
	range	48.3–219.8	0.16–3.82	0.178–11.01	0.028–0.198	0.0016–0.0223
	CV	38.7	70.0	146.8	37.4	76.5
Compact bone			*n* = 17			
	AM ± SD	108.8 ± 71.02	0.85 ± 0.51	1.016 ± 1.273	0.118 ± 0.077	0.0061 ± 0.054
	Med	67.6	0.75	0.415	0.149	0.0039
	range	60.0–278.2	0.19–2.18	0.153–4.354	0.024–0.550	0.0016–0.0226
	CV	65.3	60.6	125.3	65.0	88.7
Spongy bone			*n* = 17			
	AM ± SD	118.4 ± 47.4	0.99 ± 1.04	1.39 ± 1.853	0.149 ± 0.071	0.0031 ± 0.0022
	Med	120.4	0.56	0.564	0.190	0.0019
	range	11.7–219.5	0.01–2.75	0.096–5.993	0.035–0.226	0.0013–0.0091
	CV	40.0	104.7	133.6	47.6	70.5
Adult, *n* = 12						
Cartilage	AM ± SD	130.8 ± 61.70	0.123 ± 3.439	1.530 ± 1.564	0.123 ± 0.054	0.0045 ± 0.0049
	Med	143.6	0.82	1.124	0.144	0.0027
	range	19.6–205.4	0.104–9.45	0.165–4.827	0.031–0.184	0.0019–0.0186
	CV	47.2	148.8	102.2	43.9	108.8
Compact bone			*n* = 11			
	AM ± SD	108.4 ± 48.8	0.67 ± 0.37	0.922 ± 0.372	0.093 ± 0.068	0.0043 ± 0.0033
	Med	99.2	0.55	0.447	0.082	0.0030
	range	50.04–202.1	0.13–1.28	0.150–2.826	0.022–0.191	0.0012–0.0104
	CV	45.0	55.7	108.3	73.0	76.2
Spongy bone			*n* = 11			
	AM ± SD	100.0 ± 50.6	0.45 ± 0.33	1.654 ± 1.883	0.132 ± 0.07	0.0028 ± 0.0025
	Med	105.0	0.35	0.927	0.131	0.0020
	range	11.2–157.0	0.07–1.21	0.069–6.147	0.034–0.260	0.0013–0.0105
	CV	50.7	74.1	113.8	53.2	91.1
Immature vs. adult						
Cartilage	*U*	NS	NS	NS	NS	63.0
	*p*			0.98		0.05
Compact bone	*U*	NS	NS	NS	NS	NS
	*p*					
Spongy bone	*U*	NS	NS	NS	NS	NS
	*p*					

*U* Mann–Whitney *U*-test, *NS* statistically non-significant difference

Taking into account data from all subjects (*n* = 30), we examined the relationship between concentrations of metals present in the same kind of bone material, and between the various groups. Table 6 presents the Spearman rank correlation coefficients (*r*_s_) and significance, and the relationships concerning metals present in the same types of fox bone. The strongest synergistic relationships (*r*_s_ > 0.70) were observed in spongy bone between the Zn and Cu and Zn and Cd, as well as between Cu and Cd. A similar although a slightly weaker relationship (*r*_s_ in the range 0.50–0.70) was found in other types of bone between the Zn and Cd, and the weakest (*r*_s_ < 0.40) between concentrations of Cu and Hg in cartilage and cartilage with adjacent compact bone. Antagonistic statistically significant relationships were detected between Zn and PB, while the absolute value of *r*_s_ did not exceed 0.50 (Table 6).

**Table 6:** Spearman rank coefficient of variation of metal concentrations in the examined bone materials of the fox (*n* = 30)

Metal correlations	Cartilage	Compact bone	Cartilage with compact bone	Spongy bone
Zinc with:				
Cu	NS	NS	0.275*	0.721***
Cd	0.536**	0.688***	0.695 ***	0.828***
Pb	–0.429*	–0.451*	–0.371**	NS
Hg	NS	NS	NS	NS
Copper with:				
Pb	NS	NS	NS	NS
Cd	NS	NS	NS	0.713***
Hg	NS	0.390*	0.275*	NS
Lead with:				
Cd	NS	NS	NS	NS
Hg	NS	NS	NS	NS
Cadmium with:				
Hg	NS	NS	NS	NS

*NS* statistically non-significant

Level of significance: *p < 0.05; **p < 0.01; ***p < 0.001

The results of statistical analysis of the relationship between the concentrations of micronutrients (Zn and Cu) and highly toxic metals (Pb, Cd, Hg) occurring in various fox bone materials revealed a number of significant correlations (Tables 7 and 8).

**Table 7:** Spearman rank correlation coefficient between two microelements — Zn and Cu — determined in various type of bone material (*n* = 30) of the fox

	Zn C	Zn CB	Zn SB	Zn C + CB	Cu C	Cu CB	Cu SB	Cu C + CB
Zn C	–	0.633***	NS	0.895****	NS	NS	NS	NS
Zn CB		–	0.379*	0.882****	NS	NS	NS	NS
Zn SB			–	0.413*	NS	NS	0.721****	0.483**
Zn C + CB				–	NS	NS	NS	NS
Cu C					–	0.367*	NS	0.644***
Cu CB						–	NS	0.621****
Cu SB							–	0.389*
Cu C + CB								–

*C* cartilage, *CB* compact bone, *C + CB* cartilage with compact bone, *NS* values statistically non-significant, *SB* spongy bone

Levels of significance: *p < 0.05; **p < 0.01; ***p < 0.001; ****p < 0.0001

**Table 8:** Spearman correlation coefficients between toxic metal concentrations (Pb, Cd and Hg) in different bone materials of the fox

	Pb C	Pb CB	Pb SB	Pb C + CB	Cd C	Cd CB	Cd SB	Cd C + CB	Hg C	Hg CB	Hg SB	Hg C + CB
Pb C	–	0.887****	0.912****	0.969****	NS	NS	NS	NS	NS	NS	NS	NS
Pb CB		–	0.871****	0.927****	NS	NS	NS	NS	NS	NS	0.388*	NS
Pb C + CB			–	0.960****	NS	−0.401*	NS	NS	NS	NS	NS	NS
Pb C + CB				–	NS	NS	NS	NS	NS	NS	NS	NS
Cd C					–	0.466**	0.480**	0.730****	NS	NS	NS	NS
Cd CB						–	0.516**	0.817****	NS	NS	NS	NS
Cd SB							–	0.835****	NS	NS	NS	NS
Cd C + CB								–	NS	NS	NS	NS
Hg C									–	0.559***	0.595***	0.859****
Hg CB										–	0.517***	0.805****
Hg SB											–	0.705****
Hg C + CB												–

*C* cartilage, *CB* compact bone, *C + CB* cartilage with compact bone, *NS* statistically non-significant, *SB* spongy bone

Level of significance: *p < 0.05; **p < 0.01; ***p < 0.001; ****p < 0.0001

In the case of micronutrients, these correlations were purely synergistic, and strongest (*r*_s_ > 0.60) between Zn in cartilage and in the compact bone and samples of cartilage with adjacent compact bone, as well as between Zn in compact bone and cartilage with compact bone. Moreover, Zn concentration in the spongy bone was correlated with Zn concentration (*r*_s_ > 0.70). In the case of Cu, strong correlations (*r*_s_ > 0.60) were observed between its concentration in cartilage and cartilage with adjacent compact bone, and Cu concentration in compact bone and cartilage with adjacent compact bone (Table 7). We found a few less expressed statistically significant correlations (*r*_s_ < 0.50) between the concentration of Cu in the cartilage and compact bone, and spongy bone and cartilage with adjacent compact bone.

In the group of toxic metals, we found both positive and negative relationships between the metals present in various types of bone samples (Table 8). Lead, with the greatest affinity to bone among the studied metals, showed very strong relationships between levels determined in all types of samples. The *r*_s_ values were very large (from about 0.87 to 0.97) and highly statistically significant (*p* < 0.0001). Regression equations were calculated for the strongest relationships.

Also in the case of Cd, high values of the Spearman correlation coefficient were recorded, especially between the concentration of this metal in the cartilage with adjacent compact bone and its concentrations in cartilage, compact bone and spongy bone (*r*_s_ showed a rising trend, from 0.73 to 0.83). For the relationship between Cd concentration in the cartilage with adjacent compact bone and concentration of this metal in the spongy bone a regression equation was calculated. Also, the concentration of Hg in cartilage with compact bone strongly correlated with the cartilage, compact bone and spongy bone, albeit with a downward r_s_ trend in this sequence (from about 0.86 to 0.70). Between different metals (Table 8) we reported only two statistically significant associations (*p* < 0.05): antagonist between the concentration of Pb in spongy bone and Cd in compact bone (*r*_s_ = −0.401), and synergistic between Pb in compact bone and Hg in spongy bone (*r*_s_ = 0.388).

## Discussion

Studies on heavy metals in the environment include, among other things, measuring concentrations in tissues and organs of animals living in land and water ecosystems. In mammals, including the red and arctic fox, toxic elements are determined mostly in the liver and kidneys which perform detoxification functions.

Warm blooded vertebrates are the subject of multifaceted studies, mainly in Central Europe and especially in Poland, the Czech Republic and Slovakia (Jankovska et al. 2010; Kalisińska et al. 2011; Piskorova et al. 2003), and less frequently in the United States and Canada (Dehn et al. 2006; Hoekstra et al. 2003), i.e., in areas or decades exposed to large amounts of anthropogenic toxic substances.

In Table 9, for comparison, we summarized data from scientific literature concerning the concentrations of two micronutrients (Zn and Cu) and three toxic metals (Pb, Cd and Hg) determined in different types of bones of long-lived mammals.

**Table 9:** Concentrations of trace elements (in mg/kg) in bone material of species from order Carnivova

Species	Place	Tissue	Zn	Cu	Pb	Cd	Hg	Age	Sex	*n*	dw or ww	Source
Family - Canidae												
Red fox (*Vulpes vulpes*)	Poland	Compact bone	–	2.8	–	–	–	–	F + M	24	dw	Budis et al. 2009
	West Pomeranian province	Spongy bone		0.9	–	–	–	–				
		Cartilage	–	–	1.96	0.16	–	–	F + M	24	dw	Lanocha et al. 2009
	Spain, Donana Park		142.1	4.2	0.385	ND	0.012	im	F + M	17	dw	Millan et al. 2008
								ad				
	USA, San Diego, ZOO	Cartilage	138.0 (121.4)	–	–	–	–	im	F + M		dw (ww)	Arnhold et al. 2002
								ad				
		Spongy bone										
Domestic dog (*Canis lupus familiaris)*	Poland	Cartilage	–	–	2.28	0.13	–	–	F + M	15	dw	Lanocha et al. 2009
	West Pomeranian province	Rib	81	2.3	–	–	–	im	F + M	15	dw	Lanocha et al. 2010
		Spongy bone	80	1.8				ad				
Gray wolf (*Canis lupus)*	Canada, Yukon	Bone	–	–	2.12	–	–	im	F + M	13	dw	Gamberg and Braune 1999
								ad				
Family - Mustelidae												
European badger (*Meles meles*)	Spain, Donana Park	Bone	128.3	1.02	0.73	ND	–	im	M	1	dw	Millan et al. 2008
Family - Viverridae												
Common genet (*Genetta genetta*)	Spain, Donana Park	Bone	173.1	1.61	0.11	ND	0.023	ad	F + M	4	dw	Millan et al. 2008
Egyptian mongoose (*Herpestes ichneumon*)			145.0	0.63	0.136	ND	0.011			11		
Indian civet (*Viverra zibetha*)	India	Bone	–	–	3.9	–	25.0	–	–	10	dw	Dey et al. 1999
Family - Felidae												
Iberian lynx (*Lynx pardinus*)	Spain, Donana Park	Bone	142.1	4.2	0.385	ND	0.012	im	F + M	17	dw	Millan et al. 2008
								ad				
Leopard cat (*Felis bengalensis*)	India	Bone	–	–	22.5	–	32.2	–	–	10	dw	Dey et al. 1999
Leopard (*Panthera pardus*)			–	–	–	–	18.3	–	–			

*F* female, *M* Male, *DW* dry weight, *WW* wet weight, *ND*-not detected

In literature, there is little data on the concentrations reflecting chronic, sublethal and lethal intoxication with lead, cadmium and mercury in mammal bones. The lowest lead concentration in human bones is observed in newborns. In children aged 11 months it is 1.5 mg/kg body weight, but this concentration usually increases even dozens of times over the lifespan, and the total concentration of this element in the body of a 60- to 70-year-old male can exceed 200 mg/kg body weight (Ma 1996). The transition from a non-toxic to pathological state changes gradually, hence it is difficult to establish a clear line between non-toxic and toxic concentrations in solid elements (Kabata-Pendias and Pendias 1999). Concentrations of the three toxic elements in skeletal elements reflect chronic exposure, yet there is no proof whether the mobilization of elements accumulated in bones may occur so rapidly that it may cause the symptoms of contamination.

### Zinc and copper

In available publications, we found little data on concentrations of Zn and Cu in the cartilage and bone of Canidae. In our earlier work (Lanocha et al. 2010) on dogs from north-western Poland, Zn concentrations in cartilage and spongy bone were similar and amounted to ~80 mg/kg, and the concentration of Cu was significantly higher in cartilage than in spongy bone (2.3 and 1.8 mg/kg dw, respectively). Budis et al. (2009) examined the limb bones of foxes from north-western Poland and observed that the concentration of Cu in the compact bone was three times greater than in spongy bone (~2.8 compared to 0.9 mg/kg), but the difference between average values was not statistically confirmed. They, however, did not take into account the age of the animals as we have done in this paper. In our study, Cu concentrations in analogous materials were significantly lower, by approximately 300% and 120% in cartilage and spongy bone, respectively. Comparison of concentrations of this metal in the compact and spongy bone between two age groups (immatures and adults), showed that the Cu concentration was higher in immature foxes than in adults by 36% and 60%, respectively, but the differences between mean values were not statistically significant.

Millan et al. (2008) studied the concentrations of various heavy metals, including Zn and Cu, in the bones of the fox, the Iberian lynx (*Lynx pardinus*), common genet (*Genette genette*), Egyptian mongooses (*Herpestes ichneumon*) and badger (*Meles meles*) in Spain. These animals differed in diet, which could be one reason for the wide variation in concentrations of trace elements in their bones (Millan et al. 2008). The largest concentrations of Zn (>170 mg/kg dw) were detected in wheat-eating common genet feeding mostly on insects, small vertebrates and fish. Smaller concentrations (~130 mg/kg dw) of this metal were found in fox and badger.

The concentration of Zn in the spongy bone of foxes from Western Pomerania was ~116 mg/kg and was about 10% or so smaller than in foxes found in Spain. Copper concentration in the bones of the fox from Spain (Millan et al. 2008) was almost 20 times greater than that observed in the spongy bone from Western Pomeranian specimens. The Donana Park in Spain, from which the animals originated in the study by Millan et al. (2008), experienced an environmental disaster in 1998. Heavily polluted water from a damaged mine waste reservoir (located above the park) contaminated the park with heavy metals and penetrated its surface and ground waters. It is probable that the transfer to food chains caused a significant increase in the concentration of heavy metals, especially Cu. Similar Zn concentrations to this study were presented in the rib of a red fox from a zoo in San Diego (California, USA) (Arnhold et al. 2002).

### Lead, cadmium and mercury

Based on laboratory tests on rodents from the order Rodentia it was established that Pb concentration in bone may exceed a value of 50 mg/kg dw, which is usually associated with adverse effects of this metal on skeletal formations. This value is known as the indicator of toxic exposure. It was also found that the concentration of Pb in the hip joint bones of rodents and insectivorous small mammals (order Insectivora) from heavily polluted areas may be much larger than the aforementioned threshold value, sometimes even more than 550 mg/kg dw (Andrews et al. 1989).

Cadmium concentration in the bones of small rodents in polluted areas exceeded 3.50 mg/kg dw (Andrews et al. 1984).

In our previous studies on dog femur cartilage, Pb was ~2.3 mg/kg, and Cd concentration was significantly lower (*p* < 0.05), at 0.13 mg/kg (Lanocha et al. 2009).

In the current study, we found differences in the concentration of Pb in the cartilage, bones, compact and spongy bone between immature and adult foxes, but this was not confirmed statistically.

In comparison to the fox in the area of southern Spain (Millan et al. 2008), there was 105%, 16% and 58% higher Pb concentration in the cartilage, compact bone and spongy bone of foxes coming from the area of Szczecin, respectively, possibly due to the greater Pb contamination in that part of the country (Table 9). This can result from environmental contamination with lead, coming mainly from car fumes, as in Poland non-leaded petrol was first introduced as late as the mid-1990s. A complete ban on gasoline with toxic antiknock substances was introduced in 2005 (Directive 98/70/EC; Directive 2003/17/EC). It is estimated that each km of urban road in Poland accounted for about 8 kg of lead, and the range of its deposition beyond the road was even 100 m.

However, it should be noted that in warm-blooded vertebrates lead accumulates more intensely in the cartilage than bones, as mentioned by many researchers (Brodziak-Dopierala et al. 2009; Kalisinska et al. 2007). In the wolf from Canada (Gamberg and Braune 1999), in medium- and large-sized examples that were often shot by hunters, the concentration of Pb was similar to that recorded in the bones of the fox from Poland (Lanocha et al. 2009). The results indicate interspecies differences related to the degree of Pb contamination of the environment and the risk of intoxication with lead shot from hunters.

Cadmium was not detected in any of the bones from the studied species of wild mammals from the Donana Park, but concentrations of Pb and Hg had a high interspecies variability, related to differences in diet (Millan et al. 2008). The greatest concentration of this metal (>2 mg/kg dw) was detected in the Iberian lynx feeding mostly on rabbits which could have contained lead from shot pellets, probably the main source of lynx lead intoxication (Table 9). Much lower concentrations of lead were found in fox bones (~0.4 mg/kg dw) and the lowest was reported in common genet and Egyptian mongooses (<0.15 mg/kg dw).

In the fox from north-western Poland, Hg concentration values were an order of magnitude smaller than the amount detected in the bones of fox and wheat-eating Egyptian mongooses in the area of southern Spain (Millan et al. 2008). The highest Hg concentration was reported in wheat common genet (0.023 mg/kg dw), eating small mammals and amphibians associated with the aquatic food chain, and the smallest in the Egyptian mongoose (0.011 mg/kg dw), feeding on small invertebrates. The concentration of Hg in fox from Spain was three times higher compared to the compact bone of the fox population originating from Western Pomerania.

Dey et al. (1999) determined Hg in the bones of two species of Felidae family from north-eastern India, Bengal cat (*Felis bengalensis*) and leopard (*Panthera pardus*), and in the Indian civet (*Viverra zebitha*) from the civet family (Viverridae), which feeds on small mammals, snakes and fish. Their bones sometimes contained even over 30 mg/kg dw. In general, Hg concentrations in the bones were very small and ranged from 0.001 to 0.065 mg/kg dw (Table 9), but scientific papers on animals occasionally report concentrations of this metal that are 3–4 orders of magnitude larger (up to 30 mg/kg dw).

Although there are various studies on heavy metals in humans and wild animals, knowledge of the concentrations in bones and the effects on the osseous–articular system is still insufficient. There are many indications that the concentrations of elements in bones are strongly correlated with environmental conditions, diet and health status of various populations of people and mammals, but more and more detailed studies are required, also in Central Europe.

In addition to routine monitoring studies conducted on the abiotic environment, an urgent need exists to monitor long-term concentrations of heavy metals with long-term effect on living organisms. An important addition is provided by biomonitoring studies on domesticated and free-living mammals, including Canidae.

## Conclusion

In fox cartilage, compact bone and spongy bone, median concentrations of the metals studied could be arranged in the following series: Zn > Cu > Pb > Cd > Hg, their values ranging from 142 mg/kg to 0.002 mg/kg dw. The strongest synergistic relationships were observed in spongy bone between the Zn and Cu and Zn and Cd, as well as between Cu and Cd. Statistically significant antagonistic relationships were detected between zinc and lead in compact bone.

Bone tissue, due to its properties, may reflect levels of chronic exposure and may be the basis of indirect assessment of environmental exposure.
